# Uptake of atrial fibrillation screening aiming at stroke prevention: geo-mapping of target population and non-participation

**DOI:** 10.1186/1471-2458-13-715

**Published:** 2013-08-03

**Authors:** Johan Engdahl, Anders Holmén, Mårten Rosenqvist, Ulf Strömberg

**Affiliations:** 1 Department of Medicine, Halland Hospital, SE-301 85 Halmstad, Sweden; 2 Department of Research and Development, Halland Hospital, SE-301 85 Halmstad, Sweden; 3 Department of Clinical Science and Education, Karolinska Institute, Danderyds Hospital, Stockholm, Sweden; 4 Department of Occupational and Environmental Medicine, Lund University, Sweden

**Keywords:** Atrial fibrillation, Screening, Participation, Stroke prevention

## Abstract

**Background:**

In a screening study for silent atrial fibrillation (AF), which is a frequent source of cardiac emboli with ischemic stroke, the proportion of non-participants was considerable and their clinical profile differed from the participants’ profile. We intended to geo-map the target population and non-participation in an attempt to understand factors related to screening uptake and, thereby, obtain useful information needed to intervene for improved uptake.

**Method:**

In the municipality of Halmstad, Sweden, all residents born in 1934–1935 were invited to the screening study during April 2010 to February 2012. The total study group included 848 participants and 367 non-participants from 12 parishes. Geo-maps displaying participation, along with target-population-based geo-maps displaying proportion of immigrants and ischemic stroke incidence, were used.

**Results:**

Smoothed non-participation ratios (SmNPR) varied from 0.81 to 1.24 across different parishes (SmNRP = 1 corresponds to the expected participation based on the total study group). Among high risk individuals, the geographical variation was more pronounced (SmNPR range 0.75–1.51). Two parishes with higher share of immigrants and elevated population-based ischemic stroke incidence showed markedly lower participation, particularly among high-risk individuals.

**Conclusion:**

AF screening uptake varied evidently between parishes, particularly among high-risk individuals. Geo-mapping of target population and non-participation yielded useful information needed to intervene for improved screening uptake.

## Background

Atrial fibrillation (AF) is the most frequently encountered clinical arrhythmia. Outcome is affected among patients with AF with a higher mortality, higher risk of stroke, and higher risk for hospitalizations [1-3]. At least one fourth of stroke cases are associated with AF [4,5]. The true share of patients with stroke associated with AF is not yet known since the detection rate of AF is depending on the duration and mode of ECG monitoring [6-8]. AF is a major contributor to healthcare expenditure, particularly because of the costs for stroke care [9]. Stroke associated with AF also inflict a larger neurologic deficit and have a higher mortality [10,11].

AF can sometimes be present without symptoms, often referred to as silent AF. When silent AF is paroxysmal, diagnosis might be difficult. The actual frequency of silent AF in the population is not yet known. Silent AF is connected to an increased risk of stroke [12]. Furthermore, patients with symptomatic paroxysmal AF often have AF episodes without symptoms, making symptoms unreliable for the estimation of AF burden [13].

Oral anticoagulation (OAC) therapy is the recommended treatment for stroke prophylaxis in patients with AF and thromboembolic risk factors.

Hence, screening for silent AF seems suitable in risk populations. We (JE and MR) have initiated such a screening program aiming at stroke prevention. Results from our initial screening study have been reported elsewhere [14]. In our initial screening study, the proportion of non-participants, including those who did not respond to the screening invitation and those who actively denied participation, was considerable and their clinical profile related to stroke risk differed from the participants’ profile [14]. This called for a thorough analysis of screening uptake.

A few determinants of non-participation have reported from a two other AF screening studies. In a study in the UK including individuals over 65 years of age, problem getting to the clinic was the most frequent stated reason for not attending [15]. Clearly, there might have been various underlying factors related to this reason. Elderly persons (>75 years) were less likely to participate. We speculate that residence area played a role. In an AF screening study among 75-year old inhabitants in a Norwegian community, socioeconomic determinants of participation, such as higher income and educational level, were reported and participation was 82% [16]. Clinical profile (related to stroke risk), spatial and socioeconomic factors provide a framework of covariates to consider with respect to AF screening program participation.

Our objective was to geo-map the target population and non-participation in an attempt to understand factors related to screening uptake and, thereby, obtain useful information needed to intervene for improved uptake. In the present report we apply a novel approach, using geo-maps, to analyze participation in a community-wide screening program for AF and its geographical (neighborhood area) relation to cardiovascular risk and immigrant group status.

## Methods

### Study area and data based on target population

**Figure 1:**
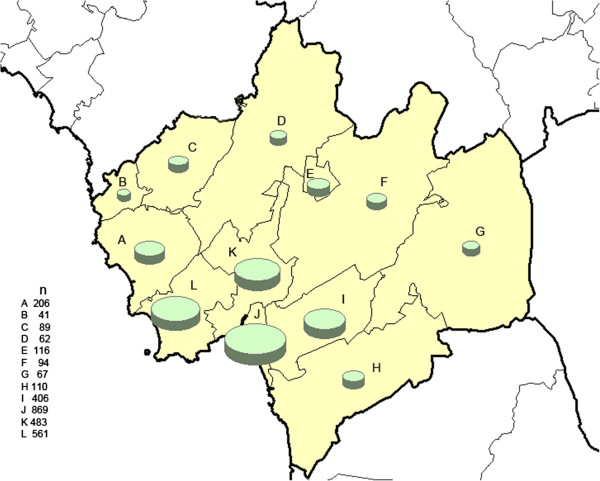
**Geo-map on population size in the age stratum 75–79 years, calendar year 2010, divided with respect to 12 parishes in the municipality of Halmstad, Sweden.** Pie size is proportional to population size.

The municipality of Halmstad is located in the southwest of Sweden and consists of 12 parishes. The total population was 91,800 inhabitants by January 1^st^ 2010. Figure 1 shows the parish-level population size for the inhabitants aged 75–79 years. On December 31^st^ 2012, the municipality had 14.6% immigrants and the five largest immigrant groups were from former Yugoslavia, Bosnia & Hercegovina, Iraq, Poland and Finland.

Statistics Sweden provided parish-level data on the proportion of immigrants among all residents aged 75–79 years. The Swedish population registry contains information on birth country, as well as residence area in Sweden, for each citizen.

Population-based data on ischemic stroke incidence were obtained for the residents aged 70+ years in the calendar period 2006–2010. For each calendar year, we stratified the population by sex and age group (70–74, 75–79, 80–84 and 85+). The ischemic stroke cases in each stratum were obtained from the regional in-patient registry (in total, n = 959). The resulting parish-specific standardized incidence ratios (SIRs) (see Statistical methods) reasonably reflect the predicted SIRs for the target population, provided unchanged impact of underlying risk factors. In other words, assuming that the target population will be followed prospectively, the SIRs provide reasonable information regarding the expected ischemic stroke risk variation in the study area. Data on stroke incidence derive from Swedish inpatient statistics, provided by The National Board of Health and Welfare. Stroke diagnosis is derived from discharge reports without conditions regarding level of care or investigations undertaken.

### Study group and individual stroke risk factors

The screening study was approved by the regional health research ethics board at Lund University, Sweden, and conducted according to the declaration of Helsinki.

All residents in Halmstad municipality born in 1934 and 1935 were invited to the AF screening program, which was performed during April 2010 to February 2012. The study invitation was sent by mail; if there was no response within 4–6 weeks, a reminder was sent. Screening visits were undertaken at a single centre at the hospital. The total study group included 848 participants (484 women) and 367 non-participants (213 women) from the 12 parishes (overall participation = 70%). We had to disregard 75 persons who actively declined study inclusion since we could not geo-code those persons, nor collect clinical data on ethical grounds. Hence, the present definition of “non-participation” refers to those who did not respond to the invitation. The absolute overall non-participation reported elsewhere [14] was therefore somewhat higher than the non-participation rates (percentages) reported in the present study.

At the index visit, all participants were asked to report their medical history including presence of AF, antithrombotic treatment and thromboembolic risk factors according to the CHADS_2_ (Congestive heart failure, Hypertension, Age > 75, Diabetes Mellitus, previous Stroke) risk classification [17]. If a patient reported a previously known diagnosis of AF, this had to be confirmed by ECG recordings in the medical records. Medical records search was made only among those participants who stated that they had an AF diagnosis and among participants with AF on ECG recordings obtained in the screening process. The accuracy of the self-reported medical history was confirmed by medical records in patients with AF. However, a random subset of 80 out of 727 patients with the questionnaire as the sole source of medical history was cross-checked against medical records in hospital and primary care, and against prescriptions. One of the 80 patients had erroneously omitted that he was treated for hypertension; in the remaining 79 cases, medical history was reported correctly.

A 12-lead ECG was recorded at index visit. We hypothesized that the diagnostic yield would be higher among patients with at least one of these risk factors, hence participants with a CHADS_2_-score of at least 2 and with no known AF after index visit were offered extended ECG recording with a hand-held event recorder (Zenicor Medical Systems, Sweden) and instructed to record 20 or 30 seconds of ECG twice daily for two weeks. Further details on the screening study are reported elsewhere [14]. Medical records from inhabitants who did not participate in the screening program were manually analysed with respect to AF diagnosis, presence of anticoagulation treatment and risk factors according to CHADS_2_. Both hospital and primary care records and prescriptions were studied. Non-participants were informed via letter and advertisement about this data collection and were thereby offered the possibility to withdraw their participation in this part of the study.

Individual data on stroke risk factor scores (CHADS_2_) were available for the 848 participants and 354 non-participants. We also analyzed data restricted to *high risk* individuals, defined as having CHADS_2_ ≥ 2.

### Statistical methods

#### Population-based data: ischemic stroke incidence

Each inhabitant was geo-coded with respect to his/her neighborhood area (parish). Geo-maps of were produced by using ESRI® ArcGIS system (Environmental Systems Research Institute, Inc., USA). The geo-map on ischemic stroke incidence was produced by computing the smoothed standardized incidence ratios (SIRs) for each parish, using the free software Rapid Inquiry Facility, which provides an extension to ESRI® ArcGIS functions [18,19]. The expected numbers of ischemic stroke episodes were obtained from the sex-, age- and calendar year-specific rates for the municipality of Halmstad (the following age groups were used: 70–74, 75–79, 80–84 and 85+). We applied the same smoothing procedure as for the participation geo-maps (see below).

#### Data from the screening study: non-participation

A parish-level non-participation ratio (NPR) was calculated as the observed-to-expected ratio, where the expected number of non-participants was obtained from the sex-specific non-participation rates for the total study group. Hence, NPR > 1 indicates *lower* participation than expected based on the data from total study group. Moreover, for each parish, a smoothed NPR (SmNPR) were obtained by running the empirical Bayesian mapping model, using a prior Gamma-model for the parish-specific non-respondent rate ratios [18,20]. More specifically, we first calculated observed-to-expected ratios (O_*i*_/E_*i*_) to estimate the parish-specific non-participation ratios (NPR_*i*_ for parish _*i*_). We applied the conventional statistical Poissonmodel for the observed numbers: O_*i*_ ~ Poisson(NPR_*i*_×E_*i*_). The empirical Bayes smoothing of the NPR_*i*_:s across the parishes was performed using a prior Gamma-model: NPR_*i*_ ~ Gamma(α, β). Such Bayesian smoothing yielded “shrinkage” of the conventional observed-to-expected ratios (NPRs) towards the expected average NPR = 1. We underline that, for a parish with few study persons, more pronounced shrinkage of an elevated/lowered NPR was obtained, as compared with a parish with a large number of study persons. Hence, by presenting smoothed geo-maps of participation, rational adjustments of the parish-specific NPRs are taken into account [19-21]: the results have higher specificity, at the price of somewhat lower sensitivity, when inferring to repeated studies. Along with a smoothed geo-map of participation, we present a statistical certainty geo-map based on the posterior probabilities of a parish-specific NPR above 1 given the data, denoted Pr(NPR>1|data).

The proportions of high risk individuals and stroke risk factors among the participants and non-participants, respectively, were compared by Fisher’s exact test. Moreover, for the high risk individuals, the CHADS_2_ scores between the participants and non-participants were compared by the Mann–Whitney test.

## Results

### Data based on target population: proportion of immigrants and ischemic stroke incidence

**Figure 2:**
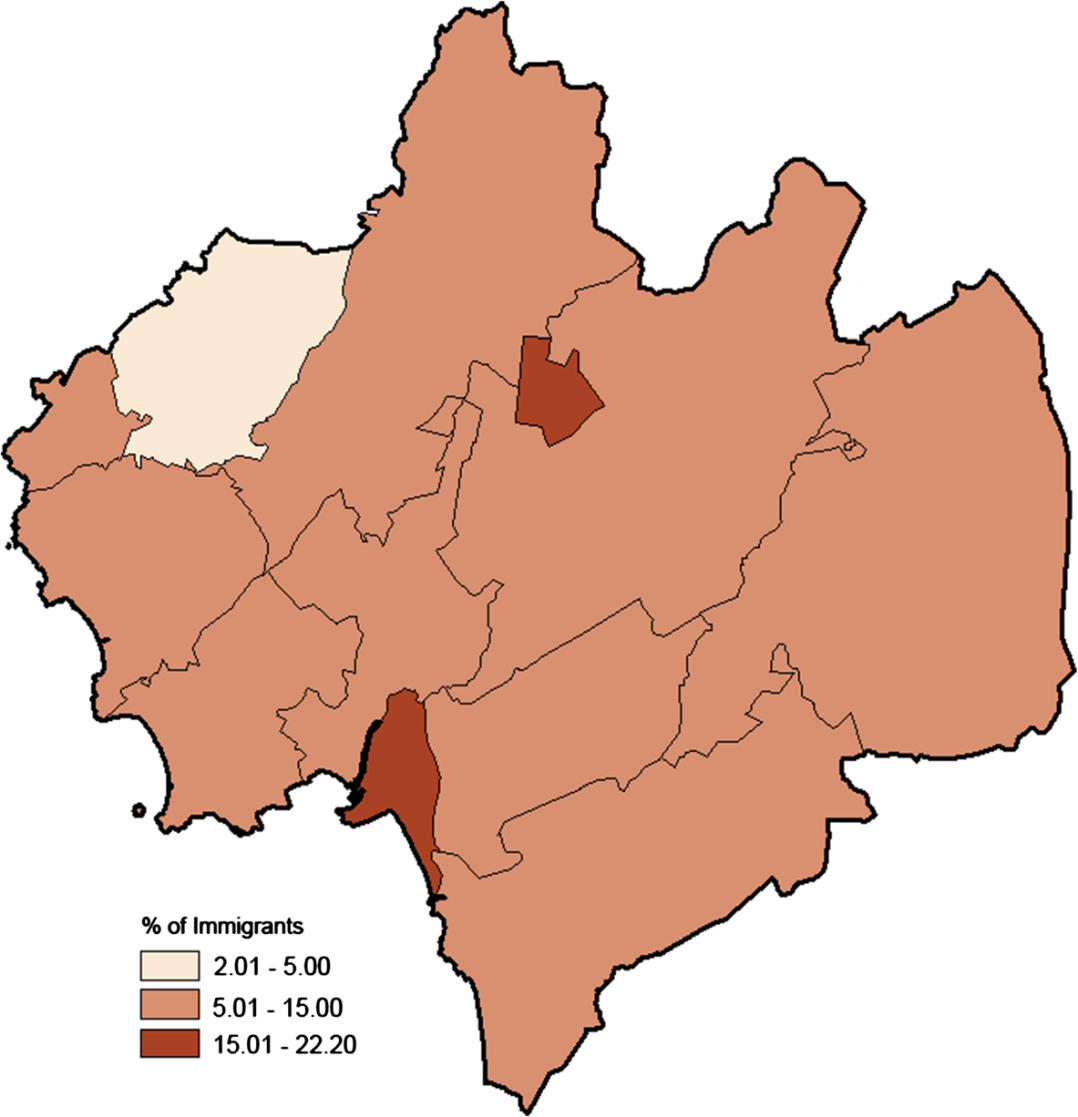
Geo-map on proportion of immigrants based on the population aged 75–79, calendar year 2010, in the municipality of Halmstad, Sweden; divided into 12 parishes (range: 2-22%).

**Figure 3:**
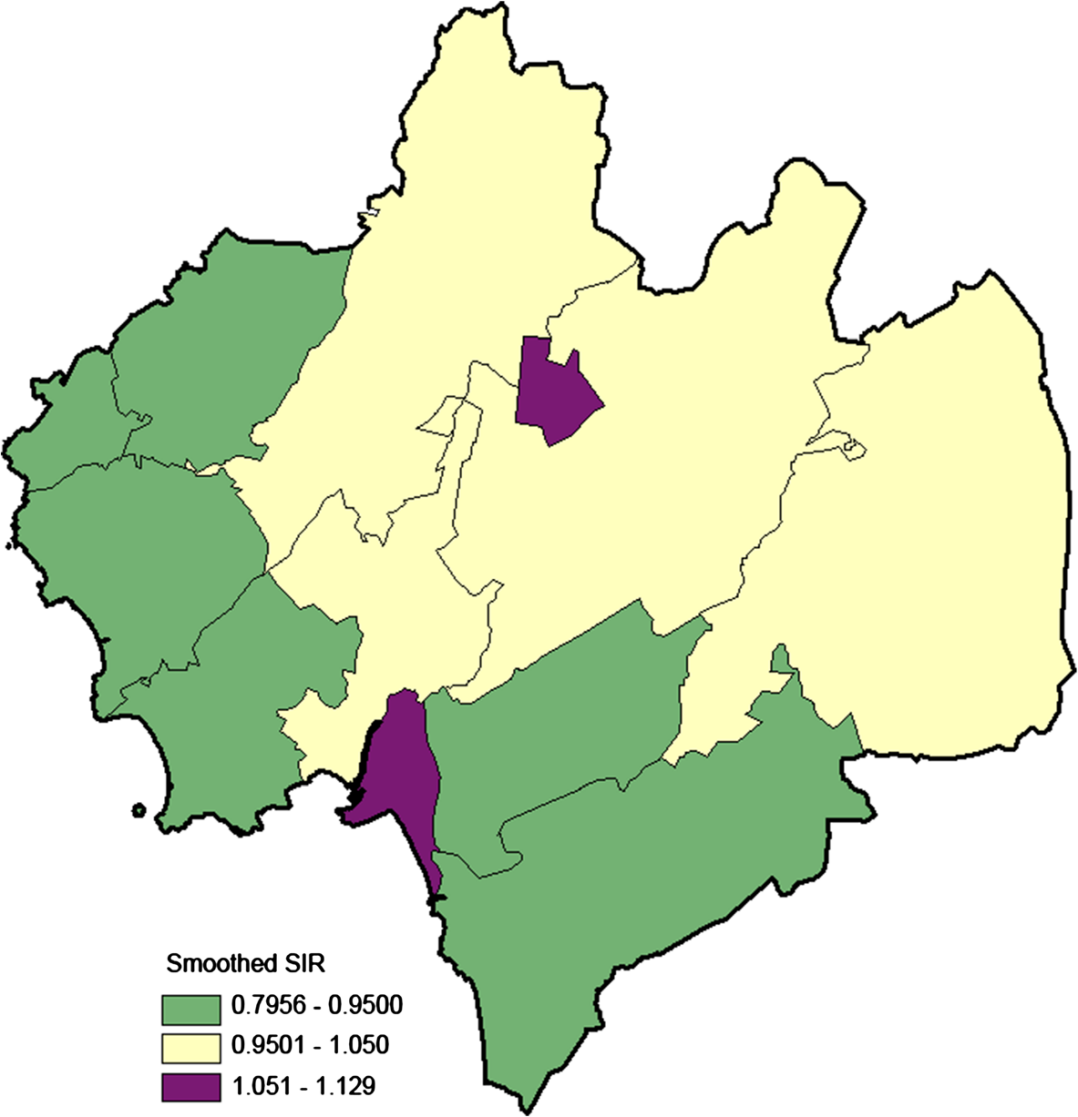
**Geo-map on ischemic stroke incidence in the population aged 70+ years, calendar period 2006–2010, in the municipality of Halmstad, Sweden; divided into 12 parishes.** The ischemic stroke cases were obtained from the regional in-patient registry (in total, n = 959). The smoothed standardized incidence ratios (SIRs) for each parish are shown (range: 0.80-1.13), which reasonably reflect the predicted SIRs for the target population, provided unchanged impact of underlying risk factors.

The proportion of immigrants among the residents aged 75–79 years varied notably across the parishes (range 2-22%; Figure 2). Also, ischemic stroke incidence in the elderly population (70+ years) varied across the parishes (smoothed SIR range: 0.80–1.13; Figure 3).

### Data from the screening study: prevalence of AF and stroke risk factors

Data on stroke risk factors was available from 352 of 367 non-participants. Non-participants more frequently had a history of heart failure, diabetes mellitus and stroke/TIA. As a consequence, their mean CHADS-score was significantly higher in comparison to participants (Table 1).

**Table 1:** Baseline characteristics including stroke risk factors among individuals attending and not attending the screening programme

**Clinical characteristics n, (%)**			
	**Participating**	**Non-participating**	
	**n=848**	**n=352**	**p**
Male gender	364 (43%)	149 (42%)	
Previously diagnosed AF	81 (9%)	39 (11%)	
Heart failure	30 (4%)	34 (10%)	<0.001
Hypertension	446 (53%)	185 (53%)	
Diabetes mellitus	91 (11%)	60 (17%)	0.004
Previous stroke/TIA	80 (9%)	49 (14%)	0.02
CHADS_2_ –score (mean)*	1.85	2.08	0.05

* CHADS_2_ –score was calculated regardless of diagnosis of AF.

### Data from the screening study: non-participation

**Figure 4:**
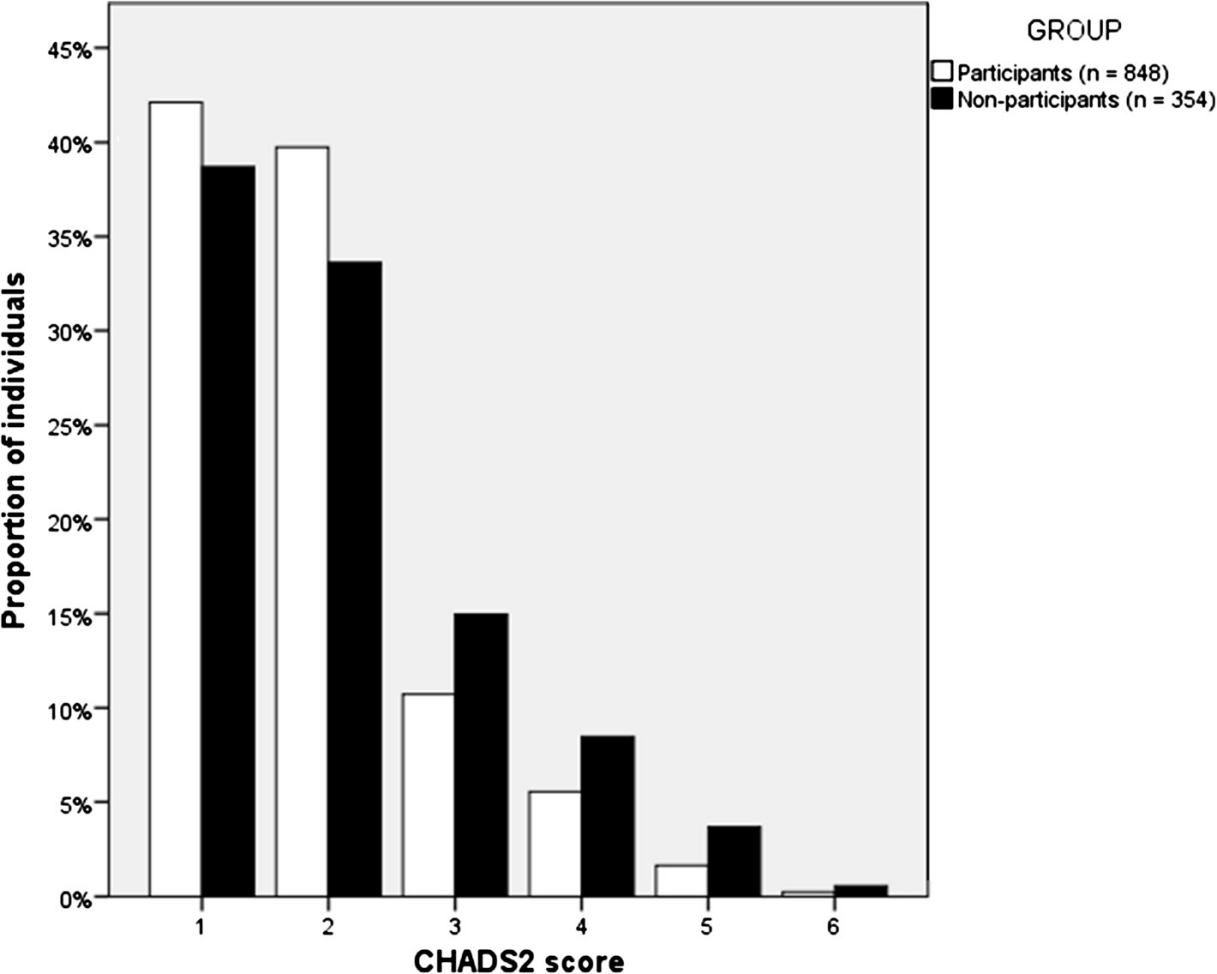
**The distribution of the individuals of the atrial fibrillation screening study according to their CHADS**_**2 **_**scores, among the participants and non-participants, respectively.**

There was no difference in participation between men and women. The proportion of high risk individuals was somewhat lower among the participants than among non-participants (491/848 = 58% vs. 217/354 = 61%, p = 0.30; Figure 4). Considering the high risk individuals, the participants had significantly lower CHADS_2_ -scores than the non-participants (mean CHADS_2_ –score 2.48 vs. 2.74, p < 0.001; Figure 4).

**Figure 5:**
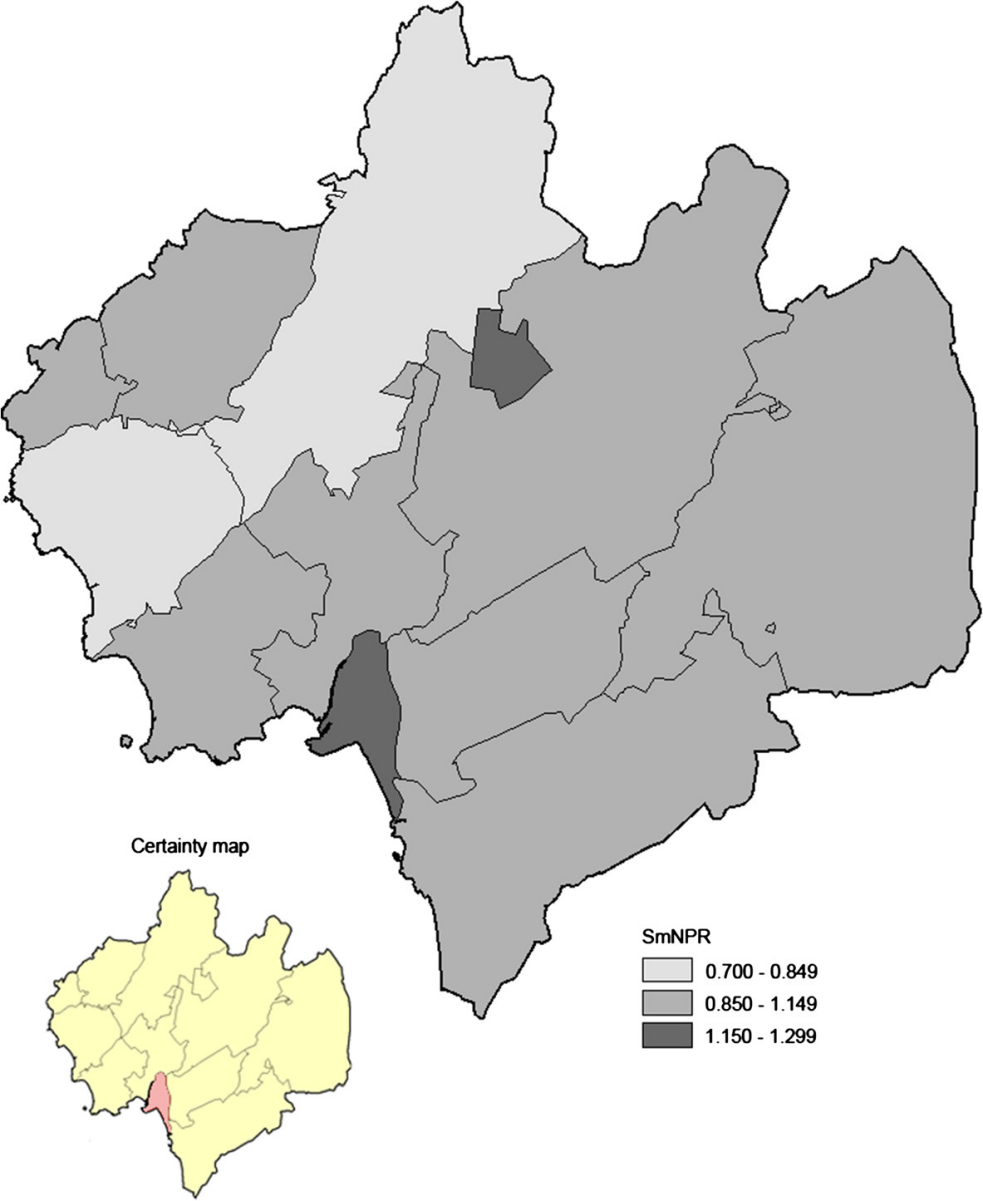
**Geo-map on participation in pilot screening study for AF, conducted in the municipality of Halmstad, Sweden; divided into 12 parishes (total study group, 848 participants and 367 non-participants).** For each parish, a smoothed non-participation ratio (SmNPR) was calculated (range: 0.81 – 1.24; SmNRP = 1 corresponds to the expected participation based on the total study group). A corresponding statistical certainty map based on the calculated posterior probabilities of a parish-specific NPR above 1 given the data [Pr(NPR>1|data)] is also shown [*light red color*, Pr(NPR>1|data) > 0.90, i.e. a parish with data yielding moderate statistical evidence of lower participation].

The parish-specific non-participation rates ranged between 18% and 48%. The smoothed geo-map based on the total study group displayed geographical variation in participation; SmNPR varied from 0.81 to 1.24 in different parishes (Figure 5).

**Figure 6:**
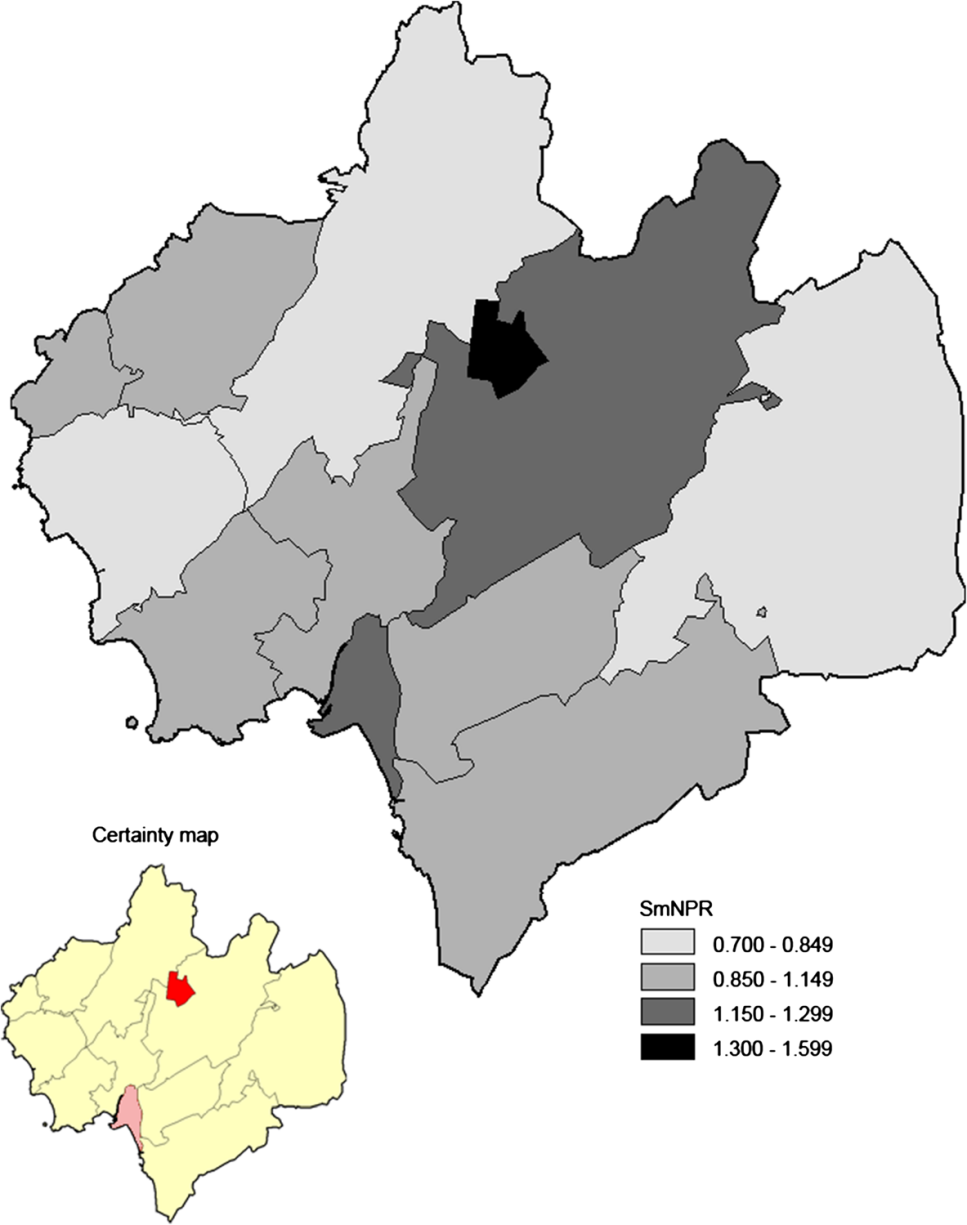
**Geo-map on participation for the high risk individuals in pilot screening study for AF, conducted in the municipality of Halmstad, Sweden; divided into 12 parishes (in total, 491 participants and 217 non-participants among the high risk indivduals).** For each parish, a smoothed non-participation ratio (SmNPR) was calculated (range: 0.75–1.51; SmNRP = 1 corresponds to the expected participation based on the total study group). A corresponding statistical certainty map based on the calculated posterior probabilities of a parish-specific NPR above 1 given the data [Pr(NPR>1|data)] is also shown [*dark red color*, Pr(NPR>1|data) > 0.95, i.e. a parish with data yielding strong statistical evidence of lower participation; *light red color*, Pr(NPR>1|data) > 0.90, i.e. a parish with data yielding moderate statistical evidence of lower participation].

Among the high risk individuals the geographical variation in participation was more pronounced. The non-respondent rates ranged between 17% and 59% and the SmNPR between 0.75 – 1.51 (Figure 6). We underline that the participation among the high risk individuals varied across the parishes. Two parishes with a relatively dense elderly population (parishes *E* and *J* in Figure 1), high proportion of immigrants (Figure 2), and elevated population-based ischemic stroke incidence (Figure 3), showed statistically evidently lower participation (Figures 5 and 6). In those two parishes, the proportions of high risk individuals were notably higher among the non-participants than among the participants (parish *E*: 16/22 = 73% vs. 11/24 = 46%; parish *J*: 75/110 = 68% vs. 119/203 = 59%). Consequently, the corresponding SmNPRs became more pronounced when considering participation among the high risk individuals (Figures 5 and 6).

## Discussion

Participation in this AF screening program varied evidently between parishes, in particular among high risk individuals, i.e. individuals that would benefit the most from anticoagulation treatment in case of an AF diagnosis. In addition, low participation was linked to higher stroke incidence and higher proportion of immigrants in the target population.

The reasons for this geographical variation could be manifold. High risk individuals might have had disabilities which affected their participation. Individuals with dementia and individuals staying at nursing homes were most probably non-responders to the screening invitation, but also in most cases not eligible to OAC treatment in case of a new AF diagnosis. A more thorough examination or interview of non-responders could have identified patients with different disabilities making participation in the screening program difficult. These data was not always available in the medical records. However, the prevalence of a previous stroke, heart failure and diabetes mellitus was significantly higher among non-respondents which would imply higher risk of disability, but data on the level of disability after stroke was not part of our data collection.

The two parishes with the lowest participation in the screening program had the highest levels of elderly immigrants among the population. Decreased ability to understand the written invitation might have impaired participation. The invitation was written in Swedish and had no condensed information or links in other languages. The participants had to schedule their index visit via a telephone call to the study center. One might speculate that this procedure affected participation and that providing the participants with a pre-scheduled appointment could increase the response rate.

In our screening study, all examinations were performed at one hospital clinic (located in parish K, see Figure 1). We speculate that problem with getting to the clinic may have affected participation in our study. In a multi-practice, cluster randomized AF screening study in the UK comparing systematic screening with opportunistic screening and no screening in routine care among individuals with age > 65 years, participation in systematic screening was 53% but varied between 22 and 68% at practice level [15]. Participation was higher among invited individuals < 75 years of age, but the yield of newly diagnosed AF was higher among those > 75 years of age. Among non-participants who stated their reason for not attending, problem with getting to the clinic was the most frequently stated reason [15]. In an AF screening study among 75-year old inhabitants in a Norwegian community reported by Tveit and coworkers, participation was 82% [16]. This study offered home visits to disabled inhabitants. It was reported that the study population had higher average income and higher education levels than national average. Both home visits and socioeconomic status might have influenced participation in this study [16].

Furthermore, our screening program was not accompanied by a media campaign.

In cancer screening settings, several factors that may influence participation have been addressed; and interventions as well as targeted actions for improving participation have been proposed [22-24].

Our results yielded useful information needed to intervene for improved screening uptake. The result points towards specific neighborhood areas for possible target actions. Yet, it might be feasible to use a modified invitation procedure for the total study group. Nevertheless, we anticipate that such an intervention will have greatest potential effect in the parishes with relatively high share of immigrants.

Although the share of immigrants and the share of non-participants not speaking Swedish fluently was unknown to us, the screening uptake might be increased by modifying the invitation letter, giving brief information and links in several languages.

Previous studies on screening for AF among elderly people have reported the significance of easy access to the screening center [15]. Performing the ECG screening closer to the participant, for instance in collaboration with primary care might increase screening uptake. As an extension of this modification, performing ECG screening via home visit in selected cases might increase screening uptake as well, although selection of these cases might prove difficult. Screening partly via home visits would also increase screening costs and would perhaps negatively influence cost effectiveness.

Participation in established screening programs in Sweden is high. For instance, 83% of invited 65-year-old men accepted to participate in aortic abdominal aneurysm screening in the Uppland region in Sweden [25]. Since our screening study was neither accompanied by a media campaign nor being part of an established screening procedure, screening uptake in a future routinely performed program might be higher than in this pilot study.

These measures will be subject to forthcoming studies. Neighborhood areas with a high proportion of immigrants among the elderly and elevated stroke incidence, which enforce screening aiming at stroke prevention, are the primary targets for action.

We have demonstrated that geo-maps on participation in our initial screening program for silent AF, along with target-population-based geo-maps on proportion of immigrants and ischemic stroke incidence, can provide valuable information in order to tailor efforts to improve participation in future screening programs.

### Limitations

Clinical and geographical data were not available from the 75 individuals who actively denied participation. If these 75 individuals were unevenly distributed among the 12 parishes, this might have altered our results slightly.

We were able to geo-code the study population into 12 different parishes. The empirical Bayes model applied means that we performed a “global” smoothing across the 12 parishes. A fully Bayseian approach provides an alternative option. By applying such an approach, a “local” smoothing is added, i.e. allowing also for dependency between rates in adjacent areas. A fully Bayesian approach is reasonable provided feasible geo-coding of the study population into several small areas. With the 12 different areas (parishes) considered, however, the fully Bayesian approach in Rapid Inquiry Facility along with free software for Bayesian data analysis, WinBugs [26] yielded an overly marked smoothing: For the total study group, those smoothed non-participation ratios varied between 0.95-1.06 across the 12 parishes and, for the high risk individuals, between 0.94-1.11.

Regarding individual-level data for the non-participants, there was limited access to socio-demographic characteristics due to ethical restrictions. In fact, the individuals of the study population could only be stratified on stroke risk factors (we used the CHADS2-score) and sex, in order the perform Bayesian smoothing of non-participation rates across the parishes. Provided richer data, geographically-weighted regression offers an interesting alternative method to identify spatial discrepancies in observed-to-expected ratios [27].

Data on thromboembolic risk factors among non-participants were collected from medical records which has certain limitations.

Our data were too spare for analyzing participation among high risk individuals defined by a more strict definition than CHADS_2_ ≥ 2.

## Conclusion

In conclusion, participation in an initial AF screening program varied notably between parishes, in particular among high risk individuals. Low participation was linked to higher stroke incidence and higher proportion of immigrants in the target population. Geo-maps on different aspects of participation provided useful information needed to intervene for improved screening uptake.

## Competing interests

Dr Engdahl has received lecture fees from AstraZeneca, Medtronic and Boehringer Ingelheim and consultant fees from Sanofi Aventis. Dr Rosenqvist has received lecture fees from Sanofi Aventis, Merck Sharpe & Dome, Bayer, Boehringer Ingelheim, Pfizer and Medtronic, consultant fees from Sanofi Aventis, Merck Sharpe & Dome, Nycomed, Bristol Meyers Squibb, Bayer, Medtronic and research grants from Sanofi Aventis, Merck Sharpe & Dome boehringer Ingelheim.

## Authors’ contributions

JE conducted the screening study, extracted data from the screening study dataset and drafted the manuscript. AH retrieved data from population and stroke registries and made statistical analyses. MR conducted the screening study and participated in study design. US contributed to study design, made statistical analysis including data for geo-maps and drafted the manuscript. All authors read and approved the final manuscript.

## Pre-publication history

The pre-publication history for this paper can be accessed here:

http://www.biomedcentral.com/1471-2458/13/715/prepub
